# The effects of moxibustion in chronic heart failure patients: a systematic review and meta-analysis

**DOI:** 10.3389/fcvm.2025.1552091

**Published:** 2025-07-15

**Authors:** Guancheng Chen, Wenxin Song, Weiwei Wu, Xuan Li, Jinyan Chen, Qiao Yang, Huili Liao

**Affiliations:** ^1^ The First Clinical Medical College of Guangzhou University of Chinese Medicine, Guangzhou, Guangdong, China; ^2^ Department of Geriatrics, The First Affiliated Hospital of Guangzhou University of Chinese Medicine, Guangzhou, Guangdong, China

**Keywords:** chronic heart failure (CHF), moxibustion, randomized controlled trials, systematic review, meta-analysis

## Abstract

**Objective:**

Moxibustion has been utilized in China for 2,000 years as a safe and straightforward intervention for chronic heart failure (CHF). Numerous articles indicate that moxibustion enhances quality of life and certain heart failure indicators in CHF patients; however, there is a deficiency of high-quality, evidence-based studies with large sample sizes. Our objective was to systematically summarize and assess the clinical efficacy of moxibustion as an adjunctive treatment for CHF.

**Methods:**

A thorough search was performed across the PubMed, Cochrane Library, Embase, Web of Science, China Knowledge Network Database, Vipers Database, Wanfang, and China Biomedical Literature Database from their inception until 1 August 2024. A meta-analysis of randomized controlled trials was utilized to aggregate the pooled metrics in patients with chronic heart failure (CHF) and to compare the clinical efficacy rate, N-terminal pro-B-type natriuretic peptide (NT-proBNP), left ventricular ejection fraction (LVEF), 6 min walk test (6MWT), and cardiac output (CO) variations between standard CHF treatment and standard treatment combined with moxibustion for CHF.

**Results:**

The study encompassed 22 randomized controlled trials (RCTs) involving 2,039 participants, with 1,021 in the experimental group and 1,018 in the control group. The experimental group exhibited a superior clinical efficacy rate compared with the control group (RR = 1.230, 95% CI: 1.173–1.289, *p* < 0.05), reduced NT-proBNP levels [standardized mean difference (SMD) = −1.035, 95% CI: −1.730 to −0.340, *p* < 0.05], enhanced LVEF (SMD = 0.909, 95% CI: 0.704–1.114, *p* < 0.001), improved 6MWT (SMD = 0.909, 95% CI: 0.704–1.114, *p* < 0.001), and increased CO (SMD = 1.0873, 95% CI: 0.882–1.293, *p* < 0.001). Following the application of funnel plots and the trim-and-fill method, the findings regarding clinical efficacy rate, LVEF, 6MWT, and CO were deemed reliable, whereas the results for NT-proBNP were found to be unstable. Subgroup analysis revealed that the number of moxibustion points contributed to heterogeneity in LVEF, 6MWT, and CO, while treatment duration accounted for heterogeneity in 6MWT.

**Conclusion:**

The study demonstrates that, in comparison with standard treatment, the integration of moxibustion for CHF patients markedly enhanced the efficacy rate, LVEF, CO, and 6MWT and may reduce NT-proBNP levels, but this result requires further validation with larger sample sizes and standardized testing methods.

**Systematic Review Registration:**

https://www.crd.york.ac.uk/PROSPERO/, PROSPERO (CRD42022372386).

## 1 Introduction

Chronic heart failure (CHF) is the result of multiple cardiovascular disorders, defined by three criteria: (1) structural and/or functional abnormalities of the heart leading to impaired ventricular filling (diastolic function) and/or ejection capacity (systolic function); (2) the manifestation of clinical symptoms and/or signs associated with heart failure; and (3) typically characterized by elevated levels of natriuretic peptides and/or imaging indicative of pulmonary or systemic congestion of cardiac origin or hemodynamic assessments suggesting impaired ventricular filling (1, 2). Recent studies indicate that approximately 6 million individuals in the United States suffer from heart failure, representing 1.8% of the entire US population, with prevalence rates between 1.5% and 1.9% in Canada and 1%–2% in Europe (3). CHF is the predominant reason for hospitalization among individuals over 65 in China, affecting over 12.1 million people aged 25 and older. The average yearly hospitalization cost for CHF patients is $4,000, constituting roughly 24.2 percent of China's per capita GDP (4). The primary objective of CHF treatment is to prevent myocardial remodeling and rehabilitate heart function. Standard pharmacological treatments comprise β-blockers, ACE inhibitors, ARBs, ARNIs, MRAs, and SGLT2 inhibitors (5). Although these treatments significantly mitigate clinical symptoms and decrease complications, their effect on recurrence rates, hospitalizations, and mortality is limited (6). A prior study (7) indicated that extended use of ACE inhibitors/ARBs may lead to a transitory deterioration in renal function and elevated blood potassium levels, along with potential transient elevations in urea nitrogen and creatinine during the early treatment stages (1). Given the constraints of current CHF medicines, research (8, 9) has investigated alternate external treatment modalities to alleviate the negative effects linked to traditional treatments. Moxibustion involves heating and fumigating body surface acupoints primarily with moxa columns or sticks. It is extensively utilized in Chinese medicine for disease prevention, treatment, and healthcare, due to its adaptability and convenience. Its main component is wormwood made from dried leaves of *Artemisia argyi*, usually processed into cylindrical moxa columns or strip-shaped moxa sticks. When used, the heat and smoke generated by burning moxa fibers stimulate acupoints on the body surface, exerting therapeutic effects. It is widely used in Chinese medicine for disease prevention, treatment, and healthcare, owing to its versatility and ease of use. As a non-pharmacological intervention, moxibustion exerts cumulative effects that develop over weeks rather than immediate short-acting responses. Recent data indicate that moxibustion, when integrated with normal CHF treatments, surpasses conventional therapy alone in enhancing left ventricular ejection fraction (LVEF), serum BNP levels, cardiac function, and myocardial ATP levels, while also diminishing myocardial fibrosis (6). There have been studies on the efficacy of traditional Chinese medicine in the treatment of CHF (10, 11). Nonetheless, these studies do not examine the application of moxibustion in isolation; instead, they concentrate on the integration of many traditional therapies with conventional treatment for CHF. There is limited data and discussion regarding moxibustion for the management of CHF. Furthermore, several of these papers lack contemporary research data. There is a deficiency of evidence-based research that systematically evaluates the effectiveness of moxibustion in the management of CHF. This study sought to evaluate the effectiveness of moxibustion in conjunction with standard treatment for CHF, assessing both clinical efficacy rates and laboratory indicators.

## 2 Methods

This study was preregistered on the PROSPERO platform (CRD42022372386) and conducted according to the Preferred Reporting Items for Systematic Reviews and Meta-analyses (PRISMA) statement (12).

### 2.1 Search strategy

The PubMed, Cochrane Library, Web of Science, and Embase electronic databases were searched for English literature; the China Knowledge Network Database (CNKI), Vipers Database (VIP), Wanfang, and China Biomedical Literature Database (CBM) were searched for Chinese literature according to the eligibility criteria from their inception to 1 August 2024, with the key terms “heart failure,” “cardiac failure,” “myocardial failure,” and “moxibustion.” The specific search strategy for PubMed is shown in Supplementary Table 1.

### 2.2 Inclusion criteria

Studies were included if they met the following:

(1) Study type is a randomized controlled trial (RCT) published in either Chinese or English.
(2) Participants met the diagnostic criteria for CHF, including both heart failure with reduced ejection fraction (HFrEF) and heart failure with preserved ejection fraction (HFpEF), with no age, sex, race, nationality, or regional restrictions.
(3) The control group underwent standard therapies, comprising ACE inhibitors, diuretics, β-blockers, aldosterone receptor antagonists, digitalis, and other medications, as well as interventions such as trigger elimination, sodium intake limitation, volume management, and oxygen therapy. The experimental group received moxibustion, which was incorporated into the therapy of the control group, with no restrictions on the method, material, application site, quantity, or duration of moxibustion.
(4) The principal outcome is clinical effectiveness rate, and the secondary outcomes include levels of N-terminal pro-B-type natriuretic peptide (NT-proBNP), left ventricular ejection fraction (LVEF), cardiac output (CO), and distance achieved in the 6 min walk test (6MWT).
(5) Protocol compliance note:​ While no universal moxibustion guidelines exist, all included studies adhered to standardized Chinese protocols (GB/T 21709.5–2021*). Interventions followed national technical specifications for acupoint selection, moxa material quality, and thermal safety controls.

### 2.3 Exclusion criteria

Studies were excluded if they met the following: (1) duplicate publications; (2) non-RCT including animal experiments, medical experience, case reports, dissertations, and reviews; (3) not containing main or secondary outcome indicators; and (4) use of other Chinese medicine-related treatments other than moxibustion in either the experimental or control group.

### 2.4 Literature selection and data extraction

Two researchers (WS and WW) separately performed literature screenings utilizing EndNote 20 software to remove duplicates, subsequently examining titles and abstracts to exclude unnecessary articles. Subsequently, they evaluated the literature according to established eligibility criteria and meticulously reviewed the complete texts to finalize inclusion judgments. Disputes were settled via dialogue or by consulting a third researcher (XL).

The principal components of the data derived from the pertinent literature encompassed the following items: authors of the included studies, year of publication, average age of patients, diagnostic criteria, sample size, intervention modality, treatment duration, clinical efficacy, NT-proBNP, LVEF, CO, 6MWT, moxibustion sites, number of moxibustion points, and adverse events (AEs).

### 2.5 Quality assessment

The quality of the included studies was evaluated using RoB2 (Version 2 of the Cochrane technique for assessing risk of bias in randomized trials), revised in 2019 (46), which encompasses six aspects: (1) randomization process; (2) deviations from intended interventions; (3) missing outcome data; (4) measurement of the outcome; (5) selection of the reported result; and (6) overall bias. The responses for items in the domains comprised “yes, Y,” “no, N,” “probably yes, PY,” “probably no, PN,” “no information, NI,” and “not applicable, NA.” The assessment was thereafter categorized into three criteria: “low risk,” “high risk,” or “some concerns.” If the bias risk assessment across all domains is classified as “low risk,” then the overall bias risk is deemed “low risk.” If the risk of bias assessment indicates “some concerns” in some areas and no areas are classified as “high risk,” the total risk of bias is deemed “some concerns.” If any area is assessed as having a “high risk” of bias, the entire risk of bias is classified as “high risk.” Two researchers (XL and JC) independently assessed the included studies, while a third researcher (QY) resolved the variance issue.

### 2.6 Statistical analysis

The R4.3.3 program was utilized for data processing and graphical representation. Effect indicators were represented as relative risk (RR) for dichotomous variables and standardized mean difference (SMD) for continuous variables, with both accompanied by 95% confidence intervals (CI) computed. Due to the fact that certain effect values in the article were derived from processed calculations and that some data mean distributions exhibit significant disparities, the standardized mean difference (SMD) was selected. The BC method (13) was employed to transform the non-normally distributed data, represented as median and quartiles, into the format of mean ± standard deviation. The mean and standard deviation of effect sizes were computed using the Cochrane User Guide, with R set at 0.5, based on the mean and standard deviation of baseline and endpoints reported in the literature. The *Q* test and *I*^2^ test assessed inter-study heterogeneity; if heterogeneity was not statistically significant (*P* > 0.1 and *I*^2^ < 50%), a fixed-effects model was employed; otherwise, a random-effects model was utilized. Funnel plots were generated, and the Egger test or Begg test was conducted to evaluate publication bias when the quantity of pooled literature reached eight or more. Furthermore, we conducted subgroup analyses based on the number of moxibustion points, treatment duration, and average patient age to investigate potential sources of heterogeneity. In the absence of possible data integration, a descriptive analysis was conducted.

## 3 Results

### 3.1 Literature selection

**Figure 1:**
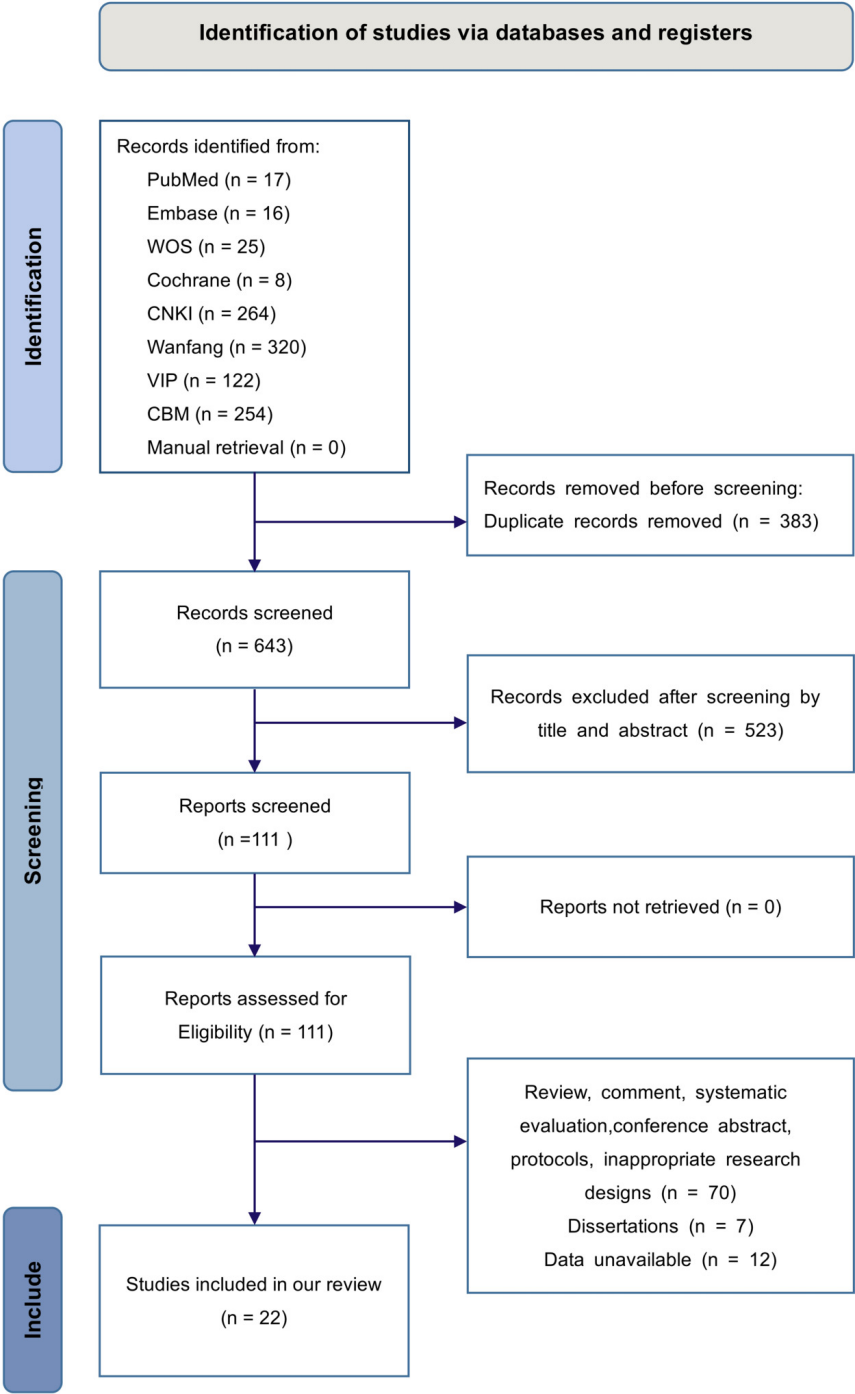
The screening process of the literature.

We acquired 1,026 studies from the initial search that rigorously complied with the established literature search technique. Subsequent to the elimination of 383 duplicate studies and the further exclusion of 523 based on irrelevant titles and abstracts, 111 studies were retained. After a thorough full-text examination, 89 papers were removed, comprising 77 that were not randomized controlled trials (RCTs) and 12 with unavailable data. As a result, we have 22 studies (14–35) included in our meta-analysis. The specifics of the literature screening are illustrated in Figure 1.

### 3.2 Characteristics and quality of included studies

The 22 included studies, all of which were RCT studies, were conducted between 2,009 and 2,023. They included 2,039 CHF patients aged 34–89 years, with 1,036 in the experimental group and 1,033 in the control group. The number of moxibustion points treated in the investigations varied from 2 to 16, and the duration of treatment ranged from 4 to 90 days. Table 1 illustrates the fundamental attributes of the studies that were incorporated.

**Table 1:** Characteristics of the included studies.

Included studies	NYHA	Number of patients included		Age (years)		Interventions		Treatment duration (days)	Outcomes	Acupoint
		CG	EG	CG	EG	CG	EG			
Jin Z, 2021	Ⅱ–Ⅳ	50	50	79.80 ± 7.34	79.72 ± 8.64	Conventional treatment	Moxibustion + C	7	①	CV4, CV6, CV8, CV12
Jie YL, 2017	Ⅰ–Ⅵ	40	40	66.9 ± 7.3	65.7 ± 8.6	Conventional treatment	Moxibustion + C	30	①③④	BL13, BL15
Yang JM, 2014	ND	26	26	77	78	Conventional treatment	Moxibustion + C	14	①	BL13, BL15
Han N, 2022	Ⅱ–Ⅳ	45	45	66.93 ± 3.27	63.77 ± 3.45	Conventional treatment	Moxibustion + C	10	①②③	CV17, BL15, BL13
Le LZ, 2020	Ⅱ–Ⅲ	40	40	69.7 ± 5.2	70.6 ± 5.0	Conventional treatment	Moxibustion + C	14	①③④	BL15, CV8, Ex-CA
Sun FJ, 2009	Ⅱ–Ⅲ	45	48	63.8	64.1	Conventional treatment	Moxibustion + C	10	①③④	CV17, CV4, CV6, ST36, BL15, BL13, BL20, BL23
Zou GH, 2011	Ⅱ–Ⅳ	30	30	46.00 ± 11.54	46.00 ± 11.54	Conventional treatment	Qiang Xin moxibustion + C	90	①③	BL15, PC6, CV4, CV9, SP9, ST36
Deng P, 2016	Ⅲ–Ⅳ	40	40	64. 38 ± 8.45	63.75 ± 10.18	Conventional treatment	Moxibustion at the heat-sensitive points + C	56	①③⑤	BL15, GV9, CV17
Zhang L, 2021	ND	30	30	61.9 ± 7.2	60.8 ± 9.4	Conventional treatment	Moxibustion at the heat-sensitive points + C	10	①③⑤	CV12, CV8, CV4, ST36, ST40
Gao C, 2020	Ⅱ–Ⅲ	61	61	69 ± 11	69 ± 11	Conventional treatment	Herb-partitioned moxibustion + C	84	①②③⑤	Shendao Bazhen point
Xia MH, 2021	Ⅱ–Ⅲ	40	40	60.8 ± 5.7	60.3 ± 5.6	Conventional treatment	Long-snake moxibustion + C	22	①③⑤	GV2–GV14
Cai XL, 2012	Ⅱ–Ⅳ	48	48	62.4 ± 4.9	61.2 ± 4.6	Conventional treatment	Thermal box moxibustion + C	14	①	RN3, RN4, RN6
Ye YZ, 2018	Ⅱ–Ⅳ	28	28	ND	ND	Conventional treatment	Thermal box moxibustion + C	14	①②	BL13, BL14, BL15
Zhao JL, 2018	ND	35	35	66.79 ± 4.62	67.23 ± 4.21	Conventional treatment	Dumai moxibustion + C	4	①②	DU14, BL13–BL28
Zhang L, 2019	ND	40	40	ND	ND	Conventional treatment	Thermal box moxibustion + C	10	①③⑤	ST40
Yi XF, 2019	Ⅱ–Ⅳ	24	24	63.3 ± 15.2	62.5 ± 14. 6	Conventional treatment	Pecking moxibustion + C	7	③⑤	RN3, RN6
Liu LP, 2022	Ⅱ–Ⅲ	30	30	70 ± 8.73	69 ± 9.32	Conventional treatment	Leihuo moxibustion + C	36	②③⑤	BL15, DU4, RN17, RN14, LU9, BL23, RN6, GB41
Deng NZ, 2023	ND	30	30	64.43 ± 6.21	73.54 ± 3.13	Conventional treatment	Thermal box moxibustion + C	28	①②③④⑤	RN8
Song LB, 2023	Ⅱ–Ⅳ	40	40	69.50 ± 8.26	70.50 ± 9.65	Conventional treatment	Herb-partitioned moxibustion + C	14	②	RN8
Zhang LJ, 2023	Ⅱ–Ⅲ	54	54	63.48 ± 11.14	65.12 ± 13.2	Conventional treatment	Leihuo moxibustion + C	15	①②③④⑤	LU11, SP1, DU4, DU9, DU10, DU11
Liu SS, 2023	Ⅲ–Ⅳ	25	25	75.20 ± 8.06	73.76 ± 7.56	Conventional treatment	Moxibustion at the heat-sensitive points + C	14	①②⑤	RN8, RN4, RN6, KL18, RN3, RN7, RN2
Wang Z, 2023	ND	30	30	66 ± 7	68 ± 7	Conventional treatment	Moxibustion + C	30	①③⑤	BL13, BL15

EG, experimental group; CG, control group; ND, no description; ① the clinical efficacy rate; ② NT-proBNP, N-terminal pro-B-type natriuretic peptide; ③ LVEF, left ventricular ejection fraction; ④ CO, cardiac output; ⑤ 6MWT, 6 min walk test.

### 3.3 Assessment of study quality and risk of bias in included trials

The risk assessment of bias for the 22 RCTs, utilizing the RoB2 with the Cochrane criteria, indicated that nine of the articles (14, 16, 17, 19, 21, 24, 29, 30, 34) failed to disclose whether the allocation sequence was concealed until participants were enrolled and assigned to interventions. Consequently, the “randomization process” was deemed to have some concerns. One study (25) indicated that blinding was implemented for the patients, whereas participants in other studies were cognizant of their treatment. The integration of moxibustion with conventional treatment for CHF presents challenges in implementing blinding. Our study indicators remained unaffected by patient subjectivity, and we employed suitable analytic methods. Consequently, the patients in the study were aware of the treatment they were receiving, which did not influence the trial results. The risk associated with “Deviations from intended interventions” was assessed to be low. All studies utilized the complete outcome data from subjects, resulting in a low risk of “missing outcome data.” The studies employed suitable outcome measures, with outcome assessors blinded to the intervention. Additionally, there was consistency in measurement and ascertainment across intervention groups, indicating a low risk in the “measurement of the outcome.” The outcome analyses across all studies aligned with the established analysis plan and were not selectively reported from various outcomes, indicating a low risk for the “Selection of the reported result.” Nine studies raised some concerns regarding “overall bias,” while 13 studies were assessed as low risk, and none were classified as high risk. The specifics regarding the assessment of risk of bias are presented in Supplementary Files 1 and 2.

### 3.4 Primary outcome

#### 3.4.1 Clinical efficacy rate

**Figure 2:**
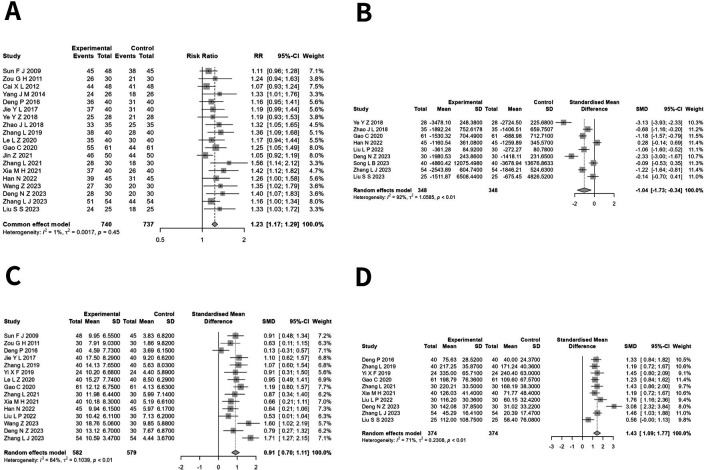
**(A)** Forest plot of risk radio in clinical efficacy rate of moxibustion in conjunction versus standard treatment in CHF. **(B)** Forest plot of standardized mean difference in NT-proBNP of moxibustion in conjunction versus standard treatment in CHF. **(C)** Forest plot of standardized mean difference in LVEF of moxibustion in conjunction versus standard treatment in CHF. **(D)** Forest plot of standardized mean difference in 6MWT of moxibustion in conjunction versus standard treatment in CHF.

Nineteen studies (14–22, 24–29, 31, 32, 34, 35) encompassing a total of 1,851 patients with CHF, with 927 participants in the experimental group and 924 in the control group, documented the clinical efficacy rate. The pooled studies exhibited minimal heterogeneity (*I*^2^ = 0.8%, *Q* = 18.15, *P* = 0.446), leading to the application of the fixed-effects model. The meta-analysis revealed a noteworthy distinction (RR = 1.230, 95% CI: 1.173–1.289, *P* < 0.05), indicating that the clinical efficacy of the experimental group was markedly superior to that of the control group (Figure 2A).

#### 3.4.2 NT-proBNP

Nine studies (20, 21, 25, 29, 30, 32–35) involving a total of 752 CHF patients, with 376 in the experimental group and 376 in the control group, reported the NT-proBNP levels. The heterogeneity across the studies was substantial (*I*^2^ = 92.3%, *Q* = 103.57, *P* < 0.001), thus necessitating the application of the random-effects model. The meta-analysis revealed a significant difference (SMD = −1.035, 95% CI: −1.730 to −0.340, *P* < 0.05), indicating that the experimental group demonstrated a markedly superior effect in reducing NT-proBNP compared with the control group (Figure 2B). After correction by the trim-and-fill method, the effect of NT-proBNP was no longer stable (SMD = −0.782, 95% CI: −1.596 to 0.032, *P* = 0.060).

#### 3.4.3 LVEF

Fifteen investigations (14, 15, 18, 19, 22–25, 27–32, 34), encompassing 1,161 CHF patients, reported LVEF levels, with 582 in the experimental group and 579 in the control group. The heterogeneity between studies was large (*I*^2^ = 64.4%, *Q* = 39.36, *P* < 0.001); hence, the random-effects model was chosen. The meta-analysis revealed a significant difference (SMD = 0.909, 95% CI: 0.704–1.114, *P* < 0.001), indicating that the test group demonstrated a much higher effect in enhancing LVEF compared with the control group (Figure 2C).

#### 3.4.4 6MWT

Ten studies (18, 22, 23, 25, 27, 28, 30, 32, 34, 35) with 748 patients with CHF, with 374 in the experimental group and 374 in the control group, reported the 6 min walk test (6MWT). The studies exhibited a moderate to high level of heterogeneity (*I*^2^ = 70.6%, *Q* = 30.62, *P* < 0.001), necessitating the application of a random-effects model. The meta-analysis revealed a significant difference (SMD = 1.430, 95% CI: 1.087–1.773, *P* < 0.001), indicating that the experimental group exhibited a markedly higher effect on boosting 6MWT values compared with the control group (Figure 2D).

#### 3.4.5 CO

**Figure 3:**
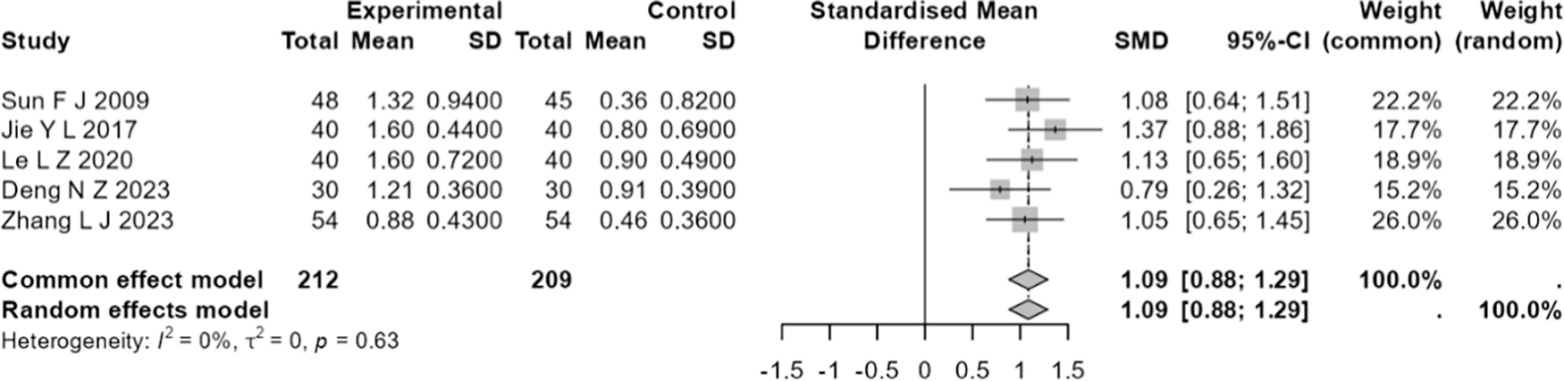
Forest plot of standardized mean difference in CO of moxibustion in conjunction versus standard treatment in CHF.

Five studies (14, 19, 24, 32, 34) involving a total of 421 patients with CHF, comprising 212 individuals in the experimental group and 209 in the control group, documented the cardiac output. The studies exhibited a degree of homogeneity (*I*^2^ < 50%, *Q* = 2.57, *P* > 0.05), thus justifying the application of the fixed-effects model. Meta-analysis showed a significant difference (SMD = 1.0873, 95% CI: 0.882–1.293, *P* < 0.001), suggesting that the effect of the test group in elevating CO was significantly better than that of the control group (Figure 3).

### 3.5 Subgroup analysis

We conducted subgroup analyses regarding clinical efficacy rates, NT-proBNP levels, LVEF, CO, and 6MWT outcomes based on treatment duration (time < 4 weeks, 4 weeks ≤ time ≤ 8 weeks, time > 8 weeks), patient age (age ≤ 65 years, age > 65 years), and the number of moxibustion points (PA < 6 points, 6 points ≤ PA ≤ 12 points, PA > 12 points). The findings from the subgroup analyses aligned with the overall results, exhibiting no shifts in direction. The variations in heterogeneity among the subgroups categorized by the number of moxibustion points indicate that the quantity of moxibustion acupoints contributed to the heterogeneity observed in LVEF, CO, and 6MWT. The heterogeneity among subgroups based on treatment duration indicates that treatment time was the underlying factor contributing to heterogeneity in the 6 min walk test (6MWT). Nonetheless, the average age of the patients did not have a significant impact on the clinical efficacy rate, NT-proBNP levels, LVEF, CO, and 6MWT outcomes. Furthermore, the length of the treatment did not significantly influence the clinical efficacy rate, NT-proBNP, LVEF, or CO. The clinical efficacy rate and NT-proBNP did not exhibit a significant correlation with the number of moxibustion points utilized. The stratification studies undertaken by various subgroups, along with the corresponding results, are presented in Table 2.

**Table 2:** Subgroup analysis of included studies.

Subgroup	Studies	Heterogeneity test results		Effect model	Meta-analysis results	
		*I* ^2^	*P*		95% CI	*P*
Clinical efficacy rate						
Time < 4 weeks	12	25%	0.2	Common	1.23 (1.16–1.31)	<0.001
4 weeks ≤ time ≤ 8 weeks	5	0%	0.54	Common	1.21 (1.11–1.33)	<0.001
Time > 8weeks	2	0%	0.95	Common	1.25 (1.07–1.45)	<0.001
Age ≤ 65 years	7	23%	0.25	Common	1.21 (1.12–1.30)	<0.001
Age > 65 years	10	0%	0.49	Common	1.24 (1.16–1.33)	<0.001
PA < 6 points	5	0%	0.86	Common	1.32 (1.18–1.47)	<0.001
6 points ≤ PA ≤ 12 points	11	0%	0.48	Common	1.19 (1.12–1.27)	<0.001
PA > 12 points	3	45%	0.16	Common	1.26 (1.12–1.41)	<0.001
NT-proBNP						
Time < 4 weeks	6	93%	<0.01	Random	−0.80 (−1.76 to 0.15)	0.1
4 weeks ≤ time ≤ 8 weeks	2	88%	<0.01	Random	−1.68 (−2.93 to −0.43)	0.008
Time > 8weeks	–	–	–	–	–	–
Age ≤ 65 years	2	93%	<0.01	Random	−0.66 (−1.77 to 0.45)	0.022
Age > 65 years	6	91%	<0.01	Random	−0.84 (−1.55 to −0.12)	0.244
PA < 6 points	2	97%	<0.01	Random	−1.20 (−3.4 to 1.00)	0.286
6 points ≤ PA ≤ 12 points	5	94%	<0.01	Random	−1.06 (−2.18 to 0.07)	0.066
PA > 12 points	2	6%	0.3	Random	−0.85 (−1.22 to −0.48)	<0.001
LVEF						
Time < 4 weeks	7	61%	0.02	Random	0.98 (0.69–1.26)	<0.001
4 weeks ≤ time ≤ 8 weeks	6	74%	<0.01	Random	0.83 (0.43–1.22)	<0.001
Time > 8weeks	2	64%	0.09	Random	0.94 (0.40–1.48)	<0.001
Age ≤ 65 years	7	77%	<0.01	Random	0.83 (0.46–1.20)	<0.001
Age > 65 years	6	41%	<0.01	Random	1.02 (0.78–1.27)	<0.001
PA < 6 points	4	27%	0.25	Random	1.12 (0.84–1.40)	<0.001
6 points ≤ PA ≤ 12 points	9	73%	<0.01	Random	0.88 (0.59–1.18)	<0.001
PA > 12 points	2	0%	0.71	Random	0.60 (0.26–0.94)	<0.001
6MWT						
Time < 4 weeks	6	32%	0.19	Random	1.22 (0.97–1.47)	<0.001
4 weeks ≤ time ≤ 8 weeks	3	86%	<0.01	Random	2.02 (1.02–3.03)	<0.001
Time > 8weeks	–	–	–	–	–	
Age ≤ 65 years	5	0%	0.94	Random	1.37 (1.14–1.59)	<0.001
Age > 65 years	3	93%	<0.01	Random	1.60 (0.15–3.05)	<0.001
PA < 6 points	2	94%	<0.01	Random	2.11 (0.27–3.96)	<0.001
6 points ≤ PA ≤ 12 points	6	33%	0.19	Random	1.25 (1.02–1.49)	<0.001
PA > 12 points	2	52%	0.15	Random	1.45 (0.89–2.00)	<0.001
CO						
Time < 4 weeks	2	0%	0.82	Common	1.08 (0.78–1.39)	<0.001
4 weeks ≤ time ≤ 8 weeks	3	20%	0.29	Common	1.09 (0.81–1.37)	<0.001
Time > 8weeks	–	–	–	–	–	–
Age ≤ 65 years	2	0%	0.93	Random	1.06 (0.77–1.36)	<0.001
Age > 65 years	3	20%	0.29	Random	1.11 (0.79–1.42)	<0.001
PA < 6 points	2	60%	0.11	Random	1.09 (0.52–1.66)	<0.001
6 points ≤ PA ≤ 12 points	2	0%	0.82	Random	1.08 (0.78–1.39)	<0.001
PA > 12 points	–	–	–	–	–	–

NT-proBNP, N-terminal pro-B-type natriuretic peptide; LVEF, left ventricular ejection fraction; 6MWT, 6 min walk test; CO, cardiac output.

### 3.6 Publication bias

We used funnel plots and Egger tests for publication bias in these indicator groups. It showed low publication because of the results of the Egger test in LVEF (*t* = 0.09, *P* = 0.926 > 0.05) and 6WMT (*t* = 1.72, *P* = 0.124 > 0.05), and the two funnel plots were relatively symmetrical. The funnel plot and Egger test of the clinical efficacy rate were performed, the funnel plots had poor symmetry, and the Egger test was significant (*t* = 6.97, *P* < 0.05), suggesting that there was publication bias. The results were corrected using the trim-and-fill method, and the correction showed a significant difference in the meta-analysis results (RR = 1.146, 95% CI: 1.092–1.204, *P* < 0.05). The symmetry of the funnel plot was elevated, and the result of the Egger test was not significant (*t* = 0.75, *P* = 0.458 > 0.05), so the results of the meta-analysis were robust. The Begg test result for the NT-proBNP group was not significant (*z* = −1.25, *P* = 0.211 > 0.05), but the funnel plot symmetry was poor, suggesting a possible publication bias. The results were corrected using the trim-and-fill method, and the correction showed that the difference in the meta-analysis results was not significant (SMD = −0.782, 95% CI: −1.596–0.032, *P* = 0.060 > 0.05), so the meta-analysis results may be unstable. Although the trim-and-fill adjustment attenuated the statistical significance of NT-proBNP reduction (*P* = 0.060), the persistent effect direction suggested a potential biological effect. This instability likely stems from methodological heterogeneity in NT-proBNP assays and unadjusted comorbidities influencing biomarker levels. Future trials should standardize NT-proBNP measurement timing and account for renal function to clarify this association. The specific analysis is detailed in Supplementary File 3.

### 3.7 Safety analysis and reporting limitations

Although moxibustion is considered safe, this review shows safety documentation gaps. Three of 22 RCTs (13.6%) reported adverse events (AEs), among which 2 (31, 35) reported no adverse effects in patients following moxibustion treatment. In a study (29) conducted on an experimental group of 45 individuals, 2 of the patients developed red papules on the epidermis in and around the moxibustion points following the moxibustion treatment. These papules resolved within 2–3 days. Two occurrences of transitory cutaneous erythema in 1,021 participants. While this shows a low prevalence of minor AEs (0.2%), the lack of standardized AE evaluation techniques across studies prevents safety judgments. But the two main subtypes of heart failure, heart failure with reduced ejection fraction (HFrEF) and heart failure with preserved ejection fraction (HFpEF), represent different pathophysiological entities that require targeted treatment strategies, which have been clearly distinguished in the European Society of Cardiology and American Heart Association guidelines. The data analyzed in this study covered patients who met the diagnostic criteria for CHF, naturally including both subtypes. However, a significant limitation is that most of the included randomized controlled trials (RCTs) did not clearly stratify results by HF subtype, nor did they systematically report the proportion of HFrEF to HFpEF patients. Therefore, this meta-analysis cannot conduct a subgroup analysis based on ejection fraction status. From a pathophysiological perspective, moxibustion may demonstrate more significant therapeutic effects in patients with HFpEF. The pathogenesis of HFpEF involves systemic inflammation, endothelial dysfunction, and the effects of comorbidities, while moxibustion may exert regulatory effects through its anti-inflammatory and microcirculation-improving mechanisms. The observed improvement in functional abilities, such as 6MWT and potential decrease in NT-proBNP levels (although the results are unstable), may be more inclined to be related to symptoms and inflammation regulation associated with HFpEF. In contrast, the core pathological mechanism of HFrEF is neurohormonal activation and ventricular remodeling. Although the effects of moxibustion on inflammation and endothelial function may also benefit HFrEF patients, the effect of moxibustion on reversing formed ventricular remodeling may be limited compared with drugs targeting neurohormone blockade. In summary, although the results of this study indicate the potential benefits of adjuvant moxibustion in the overall management of CHF, the inability to distinguish the effects of HFrEF and HFpEF subtypes remains a key limitation. Future high-quality RCTs should strictly define and stratify patients based on HF subtypes to clarify whether the therapeutic benefits of moxibustion are more significant in a specific phenotype (especially in HFpEF, where treatment options are currently more limited). What's more, other limitations include inconsistent adverse event (AE) monitoring, with 86.4% (19/22) of studies either failing to specify AE assessment methods or merely stating “no AEs observed” without detailing monitoring criteria. Additionally, potential risks such as local burns or allergic reactions to moxa smoke were not proactively investigated in any trial. No studies stratified safety data by age or comorbidity, despite the known increased dermal sensitivity in elderly CHF patients. To address these gaps, future RCTs should adhere to CONSORT Harm guidelines, incorporating standardized AE reporting with severity grading, causality assessment, and protocolized surveillance timelines.

## 4 Discussion

This appears to be the first meta-analysis focused solely on the impact of moxibustion in the treatment of CHF. In our study, the efficacy of moxibustion in combination with routine treatment in patients with CHF was better than that of routine treatment alone, as evidenced by clinical efficacy rate, NT-proBNP, LVEF, CO, and 6MWT.

The association of these five significant indicators with CHF patients is noteworthy. The articles we included assessed clinical efficacy rates using the New York Heart Association (NYHA) classification. The CHF patients were deemed to have received effective treatment if there was a reduction of at least one grade in their NYHA classification following the treatment.

Elevated NT-proBNP levels were primarily influenced by increased ventricular wall pressures, and the NT-proBNP levels measured before discharge served as significant predictors for the risk of death or hospital readmission due to CHF (36). Significant evidence indicated that NT-proBNP levels correlated with the risk of both short- and long-term adverse outcomes in heart failure patients, encompassing all-cause mortality, cardiovascular death, and major cardiovascular events (37–39).

It has been observed that while elevated NT-proBNP levels suggest left ventricular dysfunction, a significant correlation between NT-proBNP values and the extent of invasively measured myocardial dysfunction and exercise tolerance is lacking (40). Additionally, it is crucial to acknowledge that other factors may contribute to increased NT-proBNP levels in heart failure patients, such as hypertension, myocardial hypertrophy, and myocardial ischemia (41). This could account for the notable heterogeneity and inconsistent outcomes observed in our meta-analysis of NT-proBNP. The articles we included did not provide a detailed categorization of the comorbidities present in patients with CHF. Additionally, moxibustion may impact other factors that could influence NT-proBNP levels while simultaneously improving heart failure in patients, an area that warrants further investigation. NT-proBNP offers diagnostic value in patients with CHF; however, it may not reliably evaluate the severity of the patient's heart failure. LVEF and CO reflect the systolic and pumping capabilities of the left ventricle, with LVEF serving as a metric for staging heart failure. In the case of impaired diastolic function of the heart, LVEF may be normal, but the decrease in CO reflects the severity of heart failure to a certain extent, and a significant reduction in both LVEF and CO was a poor prognostic factor in patients with CHF (39, 42). The 6MWT provides a measurable assessment of cardiac functional capacity and serves as an indicator of the patient's exercise tolerance. Rostagno et al.’s study (43) indicated a correlation between the distance walked in the 6MWT and prognosis in heart failure. Specifically, patients with heart failure who walked <300 m during the test exhibited lower 3-year survival rates.

All five indicators were highly relevant to CHF patients, and moxibustion can improve these indicators, which is also consistent with previous relevant meta-analyses. In contrast to our study, which encompasses 22 articles on moxibustion for CHF, Liang et al.’s study is limited to eight articles on the subject (10), while Li et al.'s study includes only two articles (11). This indicates that our study provides a more extensive and thorough analysis of moxibustion for CHF. The improvement in these indicators due to moxibustion can be attributed to its ability to inhibit inflammation levels, promote the release of vasoactive substances, and enhance myocardial blood supply and oxygenation, subsequently leading to the inhibition of cardiomyocyte apoptosis and the enhancement of both diastolic and systolic cardiac function. This was also in line with our included studies, which showed results that the addition of moxibustion treatment reduced the level of inflammation in patients, in the RCT study involving 80 patients by Meihua et al. (28). It was found that the combination of moxibustion with conventional treatment decreased the level of galectin-3 (Gal-3) and soluble growth stimulation expressed gene 2 (sST2) more. The research conducted by Zhu et al. (31) demonstrated that moxibustion treatment can reduce the levels of myeloperoxidase (MPO) and C-reactive protein (CRP). Moxibustion at “Zusanli” (ST36) has been shown to enhance vascular endothelial integrity, counteract oxidative stress, and modulate vasodilation and contraction through the regulation of the PI3K/AKT/mTOR signaling pathway in hyperlipidemic rats (44, 45).

Moxibustion, as an external treatment modality, has the potential to enhance various indicators, activity tolerance, and symptoms in patients with CHF. Additionally, it may address complications associated with CHF, including edema. Moxibustion is understood to have the effects of warming yang to facilitate water movement, activating blood circulation, and promoting the flow of qi within the framework of traditional Chinese medicine. The studies we included indicated that conventional heart failure treatment, when combined with moxibustion therapy, has the potential to enhance diuretic effects or improve diuretic resistance. Furthermore, moxibustion therapy may reduce resistance to oral medications and increase their efficacy (22, 23, 35). Nevertheless, the available data on this aspect of studies was limited, which led us to refrain from conducting a meta-analysis. We anticipate additional data that can be examined.

Moxibustion is a gentle treatment that demonstrates a relatively high safety profile. In the 22 studies analyzed, which included a total of 1,021 participants, only 2 individuals reported mild adverse effects. The treatment is straightforward, allowing patients to perform moxibustion independently, whether in a hospital or at home. Therefore, we conclude that moxibustion warrants further investigation for its potential benefits in improving CHF.

In the subgroup analysis, although treatment duration was identified as a heterogeneity factor for the 6MWT, it did not demonstrate a significant difference in between-group heterogeneity within the NT-proBNP and LVEF groups. This finding contrasts with previous studies (11), probably because previous studies used traditional Chinese cutaneous region therapy, whereas our study focused solely on the treatment of moxibustion. We observed significant duration-dependent effects across key CHF outcomes. Neurohormonal response (NT-proBNP) peaks at 4–8 weeks (SMD = −1.68, *P* = 0.008), consistent with moxibustion's cumulative modulation of RAAS and sympathetic activation. The non-significant short-term effects (SMD = −0.80, *P* = 0.10) suggest inadequate duration for biomarker recalibration. Functional capacity (6MWT) shows maximal gain at 4–8 weeks (SMD = 2.02). This aligns with physiological timelines for peripheral vascular adaptation and skeletal muscle oxygenation. Cardiac remodeling (LVEF) exhibits early-onset benefits (SMD = 0.98 at <4 weeks) maintained through long-term treatment, potentially reflecting both acute hemodynamic effects and sustained anti-fibrotic activity. Hemodynamic improvement (CO) remains robust across durations, although the marginally higher short-term effects may relate to immediate afterload reduction. These findings support a clear intervention timeline: Short-term treatment (<4 weeks) primarily targets acute symptom relief (CO/LVEF improvement), intermediate duration (4–8 weeks) achieves optimal functional recovery (6MWT enhancement) and neurohormonal balance (NT-proBNP reduction), while extended therapy (>8 weeks) is necessary to consolidate structural benefits (CO/LVEF maintenance). However, interventions shorter than 4 weeks appear inadequate for complete biomarker recalibration, suggesting that while acute physiological responses may occur rapidly and observed effects may represent transient hemodynamic changes rather than true myocardial recovery, sustained treatment is required for meaningful cardiac remodeling and lasting clinical benefits in CHF patients. This temporal pattern reflects the progressive pathophysiological mechanisms involved in CHF management, where different therapeutic effects manifest at distinct treatment stages. Future trials should standardize intervention windows (minimum of 4 weeks) to align with CHF pathophysiology.

This study is the first examination of how the quantity of moxibustion points influences moxibustion treatment for CHF, identified as the source of heterogeneity in LVEF, CO, and 6MWT through subgroup analysis. When considering previous treatment data, an increased number of moxibustion points could potentially enhance the effectiveness of moxibustion treatment. However, additional research is necessary to establish the optimal number of moxibustion points required to maximize efficacy in patients with CHF.

In all subgroups, age did not contribute to heterogeneity, constrained by the predominance of elderly hospitalized CHF patients, primarily around 65 years old.

The age distribution within the subgroup analyses was thus more concentrated and uneven, potentially influencing the results. Expanding the sample size to achieve a more balanced age distribution among patients receiving moxibustion treatment for CHF may further clarify the impact of age on treatment efficacy.

The advantages of our present investigation are dual in nature. We are the first to investigate the efficacy of moxibustion treatment for patients with CHF in isolation, rather than in conjunction with other traditional Chinese medicine therapies. Additionally, we are the pioneers in proposing the influence of the number of acupuncture sites on the treatment of CHF patients using moxibustion. Nonetheless, the limitations of our study warrant discussion, as our study population was confined to a specific region of China, and there was a lack of data regarding treatment outcomes in other countries, rendering the efficacy of moxibustion treatment across other ethnic groups ambiguous. Lastly, the age distribution of our included population was both dense and uneven, and the implementation of blinding and the number of inclusions may be enhanced, potentially affecting our results.

## 5 Conclusion

Moxibustion is a safe and effective treatment for CHF, and it is currently used as a routine treatment in the inpatient units of Chinese hospitals. Current evidence suggests moxibustion is well-tolerated in CHF patients, with rare transient adverse events reported. However, standardized safety assessment protocols are urgently needed to establish robust safety profiles across diverse populations. While moxibustion demonstrates potential benefits across varying treatment durations, its role in long-term cardiac remodeling requires protocols ≥8 weeks. Shorter interventions (≤2 weeks) may primarily alleviate symptoms rather than modify disease progression.

Our study shows that moxibustion therapy can be added to any treatment of CHF patients to improve the efficacy. However, further multiethnic, high-quality, large-sample RCTs are necessary to confirm the efficacy of moxibustion.

## Data Availability

The original contributions presented in the study are included in the article/Supplementary Material, further inquiries can be directed to the corresponding author.

## Supplementary material

Supplementary File 1 Risk of bias assessment.

Supplementary File 3 Publication bias assessment.

## References

[1] McDonagh TA Metra M Adamo M Gardner RS Baumbach A Böhm M 2021 ESC guidelines for the diagnosis and treatment of acute and chronic heart failure. Eur Heart J. (2021) 42(36):3599–726. 10.1093/eurheartj/ehab368 34447992

[2] Tsutsui H Ide T Ito H Kihara Y Kinugawa K Kinugawa S JCS/JHFS 2021 guideline focused update on diagnosis and treatment of acute and chronic heart failure. Circ J. (2021) 85(12):2252–91. 10.1253/circj.CJ-21-0431 34588392

[3] Roger VL. Epidemiology of heart failure: a contemporary perspective. Circ Res. (2021) 128(10):1421–34. 10.1161/CIRCRESAHA.121.318172 33983838

[4] Chinese Society of Geriatrics, Electrocardiology and Cardiac Function Branch, China Heart Failure Center Alliance Expert Committee, Editorial Board of Chinese Journal of General Practitioner, Chinese Medical Association. Chinese expert consensus on early screening and primary prevention of heart failure (2024). Chin J Fam Med. (2024) 23(01):7–18. 10.3760/cma.j.cn114798-20230806-00043

[5] Greene SJ Butler J Metra M. Another reason to embrace quadruple medical therapy for heart failure: medications enabling tolerance of each other. Eur J Heart Fail. (2021) 23(9):1525–8. 10.1002/ejhf.2301 34263507

[6] Wang W Li QL Ma Q Xia R Gao B Wang Y Effect of moxibustion combined with benazepril on expression of IL-18 and phosphorylated protein kinase B in myocardial tissue of rats with chronic heart failure. Zhen Ci Yan Jiu. (2021) 46(11):935–41. 10.13702/j.1000-0607.201219 34865330

[7] Natale P Palmer SC Ruospo M Saglimbene VM Strippoli GFM. Potassium binders for chronic hyperkalaemia in people with chronic kidney disease. Cochrane Database Syst Rev. (2020) 6(6):CD013165. 10.1002/14651858.CD013165.pub2 32588430 PMC7386867

[8] Kristen AV Schuhmacher B Strych K Lossnitzer D Friederich HC Hilbel T Acupuncture improves exercise tolerance of patients with heart failure: a placebo-controlled pilot study. Heart (British Cardiac Society). (2010) 96(17):1396–400. 10.1136/hrt.2009.187930 20554511

[9] Painovich J Longhurst J. Integrating acupuncture into the cardiology clinic: can it play a role? Sheng Li Xue Bao: [Acta Physiologica Sinica]. (2015) 67(1):19–31. 25672623

[10] Liang B Yan C Zhang L Yang Z Wang L Xian S The effect of acupuncture and moxibustion on heart function in heart failure patients: a systematic review and meta-analysis. Evid Based Complement Alternat Med. (2019) 2019:6074967. 10.1155/2019/6074967 31772597 PMC6854931

[11] Li M Li H Liu H Lai X Xing W Shang J. Effect of traditional Chinese medicine cutaneous regions therapy as adjuvant treatment of chronic heart failure: a systematic review and meta-analysis. Heliyon. (2023) 9(5):e16012. 10.1016/j.heliyon.2023.e16012 37206004 PMC10189498

[12] Page MJ McKenzie JE Bossuyt PM Boutron I Hoffmann TC Mulrow CD The PRISMA 2020 statement: an updated guideline for reporting systematic reviews. Br Med J. (2021) 372:n71. 10.1136/bmj.n71 33782057 PMC8005924

[13] McGrath S Zhao X Steele R Thombs BD Benedetti A, DEPRESsion Screening Data (DEPRESSD) Collaboration. Estimating the sample mean and standard deviation from commonly reported quantiles in meta-analysis. Stat Methods Med Res. (2020) 29(9):2520–37. 10.1177/0962280219889080 32292115 PMC7390706

[14] Fujun S Huawei M Li C Changling X Xia L. Clinical observation of moxibustion combined with beta-blocker in the treatment of chronic congestive heart failure. Hebei Trad Chin Med. (2009) 31(10):1516–7. 10.3969/j.issn.1002-2619.2009.10.054

[15] Guohui Z Zhongyong L Nana T Lin L. Clinical study of strong heart moxibustion on ventricular remodeling in patients with heart failure. Jiangxi Provincial Society of Integrative Chinese and Western Medicine. Proceedings of the Ninth Symposium of Jiangxi Society of Integrative Chinese and Western Medicine on New Progress in the Clinical Application of Activating Blood and Removing Blood Stasis (2011). p. 202–6

[16] Xinlun C Guihui W. Clinical observation on 48 cases of heart failure treated by box moxibustion. Pract Chin Western Med Clin. (2012) 12(06):56–7. 10.3969/j.issn.1671-4040.2012.06.036

[17] Jinmei Y. Nursing experience of moxibustion in the treatment of chronic heart failure in the elderly. Pract Chin Western Med Clin. (2014) 14(07):80–1. 10.13638/j.issn.1671-4040.2014.07.055

[18] Peng D Dan H Zhongyong L Lin L Yang X. Clinical efficacy study of thermal moxibustion on chronic heart failure. Chin Med Bull. (2016) 15(04):37–9. 10.14046/j.cnki.zyytb2002.2016.04.015

[19] Yanli R Lijie L. Clinical observation on the treatment of chronic heart failure by moxibustion of lung yu and heart yu. Modern Nutrition. (2017) (14):1. 10.3969/j.issn.1671-0223.2017.07.149

[20] Yuzhu Y Jing Y. Clinical efficacy observation of moxibustion in treating qi deficiency and blood stasis type chronic heart failure. 2018 Annual Meeting of Nursing Branch of Zhejiang Society of Traditional Chinese Medicine. Zhejiang Society of Traditional Chinese Medicine (2018).

[21] Jinling Z Xinmei Q. Treatment of 35 cases of heart failure with chronic pulmonary heart disease by dugu moxibustion. Heilongjiang Trad Chin Med. (2018) 47(02):73–4. http://d.wanfangdata.com.cn.https.zjlib.proxy.zyproxy.zjlib.cn/periodical/ChVQZXJpb2RpY2FsQ0hJMjAyNTA2MjISD2hsanp5eTIwMTgwMjA0MRoIeXIxbXc1ZXA%3D

[22] Lan Z Haiyan J Bin L Xiaoling H Zhun Y. Clinical nursing intervention of moxibustion on edema symptoms in heart failure (cardiac and renal yang deficiency with stasis and water obstruction syndrome). Wisdom Health. (2019) 5(27):71–3. 10.19335/j.cnki.2096-1219.2019.27.036

[23] Xuefeng Y Xiaodong R. Evaluation of diuretic effect and therapeutic efficacy of bird pecking moxibustion on heart failure patients with yang deficiency and blood stasis type. Inner Mongolia Trad Chin Med. (2019) 38(04):81–2. 10.16040/j.cnki.cn15-1101.2019.04.053

[24] Lizhen L Pingping H Ruiting W Yuehong L Jinxia Y Yanping L Observation on the effect of nursing intervention of heart-care moxibustion in patients with chronic heart failure. Massage Rehab Med. (2020) 11(23):92–4. 10.19787/j.issn.1008-1879.2020.23.031

[25] Chen G Wei F Haili L Weili Z Jie L Shuxin L Observations on the efficacy of spaced-medicine moxibustion at shendao bazhen points combined with western medicines in the treatment of cardiopulmonary qi deficiency syndrome of chronic heart failure. Shanghai J Acupunct Moxibustion. (2020) 39(09):1089–93. 10.13460/j.issn.1005-0957.2020.09.1089

[26] Zhou J Xin L Junjun F. Effects of moxibustion on cardiac function, BNP and CA125 in patients with heart failure with qi deficiency and blood stasis. Zhejiang J Trad Chin Med. (2021) 56(02):134–5. 10.13633/j.cnki.zjtcm.2021.02.036

[27] Lan Z Haiyan J Xiaoling H. Heat-sensitive group acupoint moxibustion technique for the treatment of chronic heart failure. J Changchun Univ Trad Chin Med. (2021) 37(03):571–3. 10.13463/j.cnki.cczyy.2021.03.026

[28] Meihua X Jinliang L Na H. Clinical study of long-snake moxibustion combined with western medicine in the treatment of chronic heart failure with heart and kidney yang deficiency. J Acupunct Tuina. (2021) 19(4):7. 10.1007/s11726-021-1259-3

[29] Ning H. Clinical observation on 45 cases of chronic heart failure adjuvanted by moxa moxibustion. Drug Eval. (2022) 19(10):616–8. 10.19939/j.cnki.1672-2809.2022.10.12

[30] Liping L Yuqun Z Shaokuan H Xiongli F. Application of thunder fire moxibustion in the rehabilitation of patients with chronic heart failure. Wisdom Health. (2022) 8(32):159–62. 10.19335/j.cnki.2096-1219.2022.32.037

[31] Zhu W Ran X Bing G Meng Z Heng W Lingji L Observations on the efficacy of acupuncture and moxibustion with medication in treating chronic heart failure of qi deficiency and blood stasis type. Shanghai J Acupunct Moxibustion. (2023) 42(08):797–801. 10.13460/j.issn.1005-0957.2023.13.004

[32] Nengzong D. Clinical observation of moxibustion shenque in the treatment of coronary heart disease and heart failure. J Jiangxi Univ Trad Chin Med. (2023) 35(02):70–2. http://d.wanfangdata.com.cn.https.zjlib.proxy.zyproxy.zjlib.cn/periodical/ChVQZXJpb2RpY2FsQ0hJMjAyNTA2MjISEWp4enl4eXhiMjAyMzAyMDIwGghrMjV3ZWE3Zg%3D%3D

[33] Libo S Haiyan L Lili Y Xuejun L Xiaoyan C Jianqing J Evaluation of umbilical cord therapy with spaced ginger moxibustion in chronic heart failure care based on the theory of spring-summer yang nourishment. Tianjin Trad Chin Med. (2023) 40(03):345–9. 10.11656/j.issn.1672-1519.2023.03.12

[34] Lijuan Z Lixian L Ruirong Z. Observation on the efficacy of thunder fire moxibustion in the treatment of chronic heart failure. J Ext Treat Trad Chin Med. (2023) 32(06):88–90. 10.3969/j.issn.1006-978X.2023.06.035

[35] Shanshan L Yanbin L Heng S Zhangsheng C. Clinical efficacy of heat-sensitive moxibustion in the treatment of heart failure with diuretic resistance. J Jiangxi Univ Trad Chin Med. (2023) 35(01):65–8. http://d.wanfangdata.com.cn.https.zjlib.proxy.zyproxy.zjlib.cn/periodical/ChVQZXJpb2RpY2FsQ0hJMjAyNTA2MjISEWp4enl4eXhiMjAyMzAxMDE5GghsOW56bno5aw%3D%3D

[36] Mori Y Fukuma S Yamaji K Mizuno A Kondo N Inoue K. Machine learning-based prediction of elevated N terminal pro brain natriuretic peptide among US general population. ESC Heart Fail. (2024) 12(20):859–68. 10.1002/ehf2.15056 39558857 PMC11911594

[37] Santaguida PL Don-Wauchope AC Ali U Oremus M Brown JA Bustamam A Incremental value of natriuretic peptide measurement in acute decompensated heart failure (ADHF): a systematic review. Heart Fail Rev. (2014) 19(4):507–19. 10.1007/s10741-014-9444-9 25052418

[38] Fonarow GC Peacock WF Phillips CO Givertz MM Lopatin M, ADHERE Scientific Advisory Committee and Investigators. Admission B-type natriuretic peptide levels and in-hospital mortality in acute decompensated heart failure. J Am Coll Cardiol. (2007) 49(19):1943–50. 10.1016/j.jacc.2007.02.037 17498579

[39] Heidenreich PA Bozkurt B Aguilar D Allen LA Byun JJ Colvin MM Correction to: 2022 AHA/ACC/HFSA guideline for the management of heart failure: a report of the American College of Cardiology/American Heart Association joint committee on clinical practice guidelines. Circulation. (2023) 147(14):e674. 10.1161/CIR.0000000000001142 37011077

[40] Parovic M Okwose NC Bailey K Velicki L Fras Z Seferovic PM NT-proBNP is a weak indicator of cardiac function and haemodynamic response to exercise in chronic heart failure. ESC Heart Fail. (2019) 6(2):449–54. 10.1002/ehf2.12424 30788904 PMC6437429

[41] Gremmler B Kunert M Schleiting H Kisters K Ulbricht LJ. Relation between N-terminal pro-brain natriuretic peptide values and invasively measured left ventricular hemodynamic indices. Exp Clin Cardiol. (2003) 8(2):91–4. 19641656 PMC2716205

[42] Yildiz O Aslan G Demirozu ZT Yenigun CD Yazicioglu N. Evaluation of resting cardiac power output as a prognostic factor in patients with advanced heart failure. Am J Cardiol. (2017) 120(6):973–9. 10.1016/j.amjcard.2017.06.028 28739034

[43] Rostagno C Olivo G Comeglio M Boddi V Banchelli M Galanti G Prognostic value of 6-minute walk corridor test in patients with mild to moderate heart failure: comparison with other methods of functional evaluation. Eur J Heart Fail. (2003) 5(3):247–52. 10.1016/s1388-9842(02)00244-1 12798821

[44] Yanyan Y Yue Y Jie J. Acupuncture preconditioning attenuates inflammatory response in cerebral ischemia-reperfusion injured rats by modulating PI3K/AKT/mTOR signaling pathway. Acupuncture Research. (2024) 49(03):238–46. 10.13702/j.1000-0607.20230267 38500320

[45] Chengxuan L Qian X Rulin Y Haibin Z Huanxi W Jianbin Z. Improvement of oxidative stress in vascular endothelium of hyperlipidemic rats by moxibustion at 45℃. Acupunct Res. (2023) 48(04):331–8. 10.13702/j.1000-0607.2022

[46] Higgins JPT Thomas J Chandler J Cumpston M Li T Page MJ Chapter 8: assessing risk of bias in a randomized trial. In: Higgins JPT Thomas J Chandler J, editors. Cochrane Handbook for Systematic Reviews of Interventions Version 6.0 (updated July 2019). The Cochrane Collaboration (2019). Available online at: https://training.cochrane.org/handbook/archive/v6/chapter-08/

