# Supplementary figures and images for: The effects of moxibustion in chronic heart failure patients: a systematic review and meta-analysis
Source: Front Cardiovasc Med. 2025 Jul 15;12:1552091. doi: 10.3389/fcvm.2025.1552091 (PMC12303964; doi:10.3389/fcvm.2025.1552091)

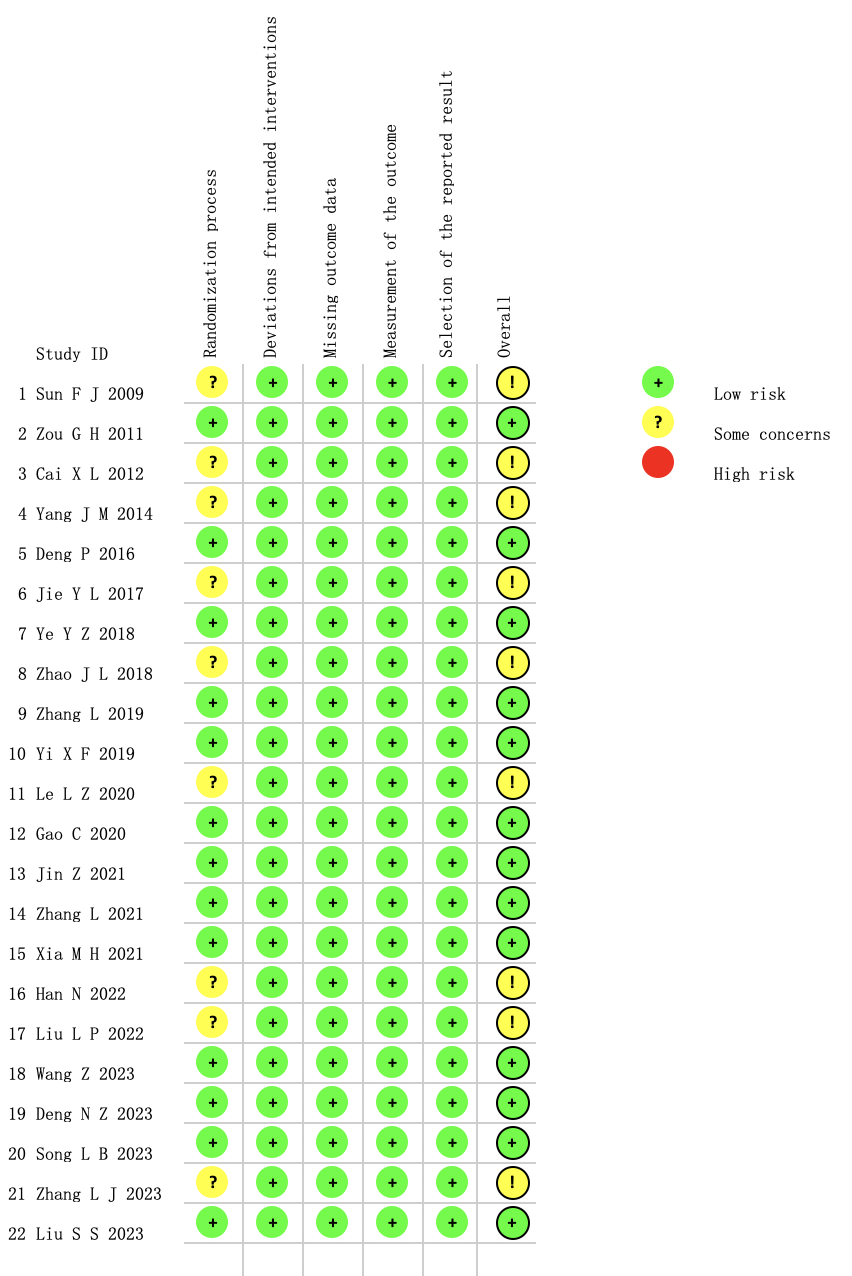

Supplement: Supplementary File 1 — Risk of bias assessment. [file Image1.jpeg]

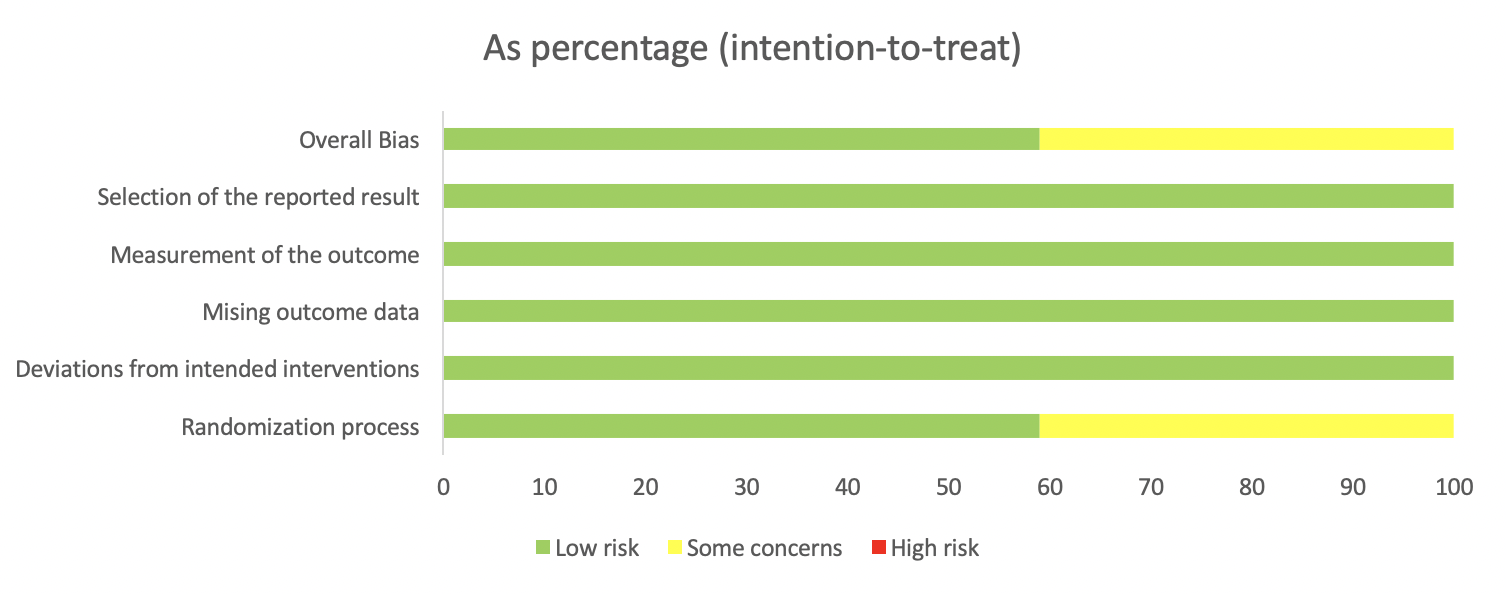

Supplement: Supplementary File 3 — Publication bias assessment. [file Image2.jpeg]
