# Supplementary material for: The effects of moxibustion in chronic heart failure patients: a systematic review and meta-analysis
Source: Front Cardiovasc Med. 2025 Jul 15;12:1552091. doi: 10.3389/fcvm.2025.1552091 (PMC12303964; doi:10.3389/fcvm.2025.1552091)
Supplement: Supplementary file 3 [file Datasheet1.docx]

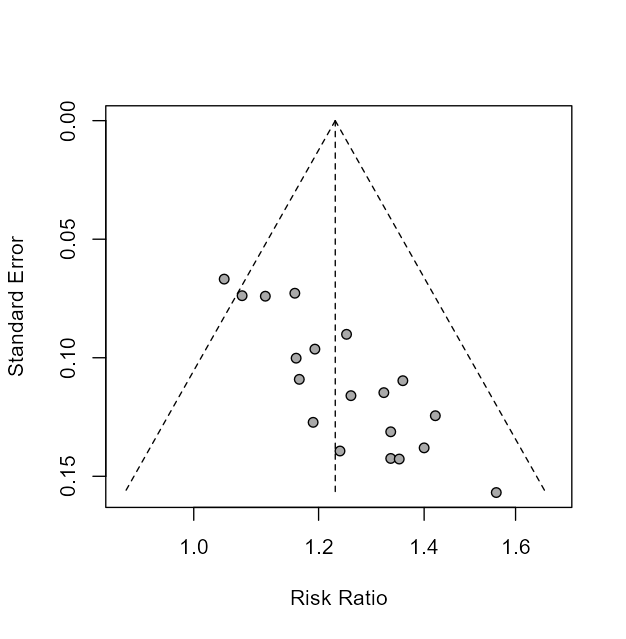


funnel plot of the clinical efficacy rate


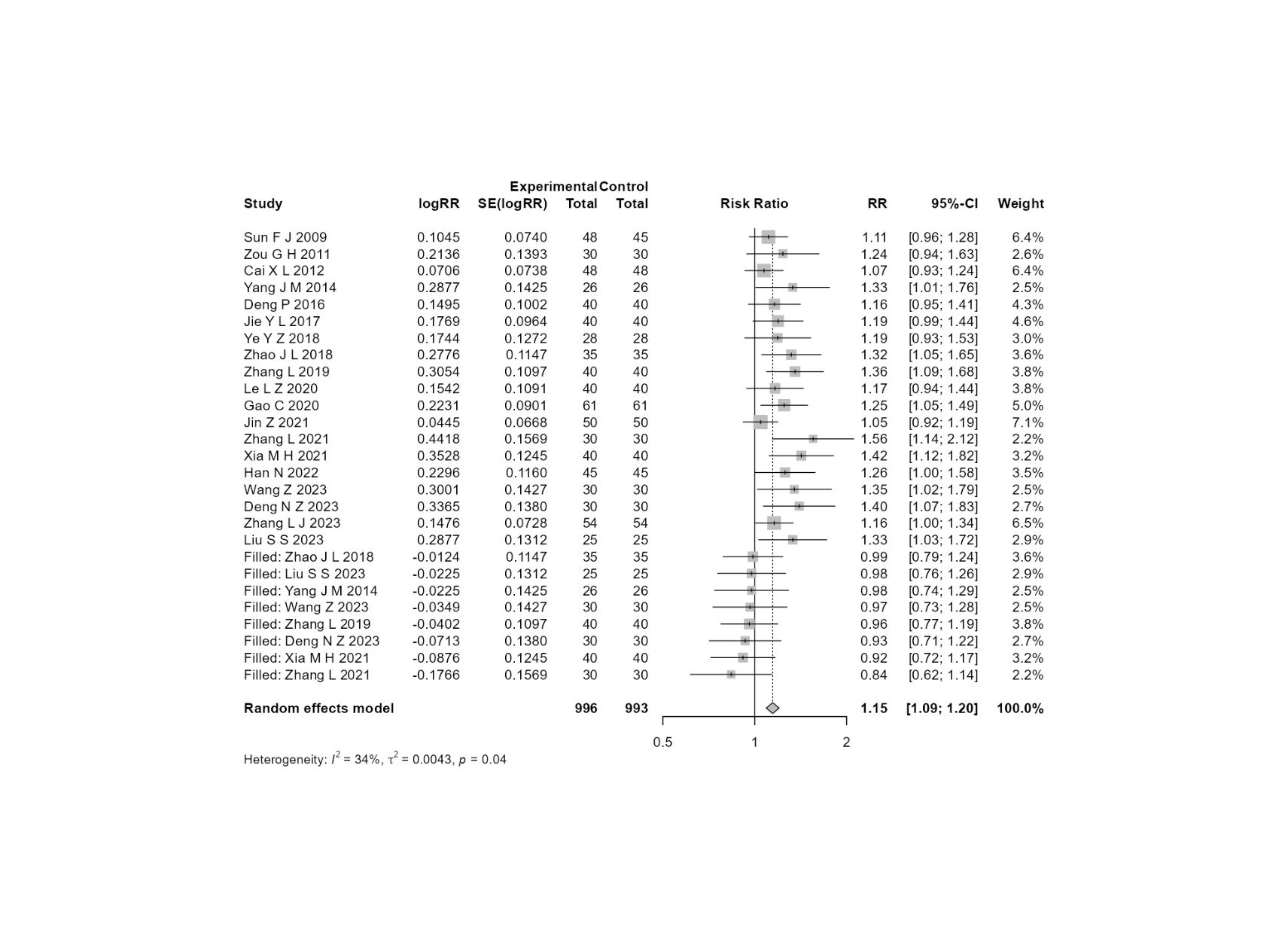


trim-and-fill method for the clinical efficacy rate


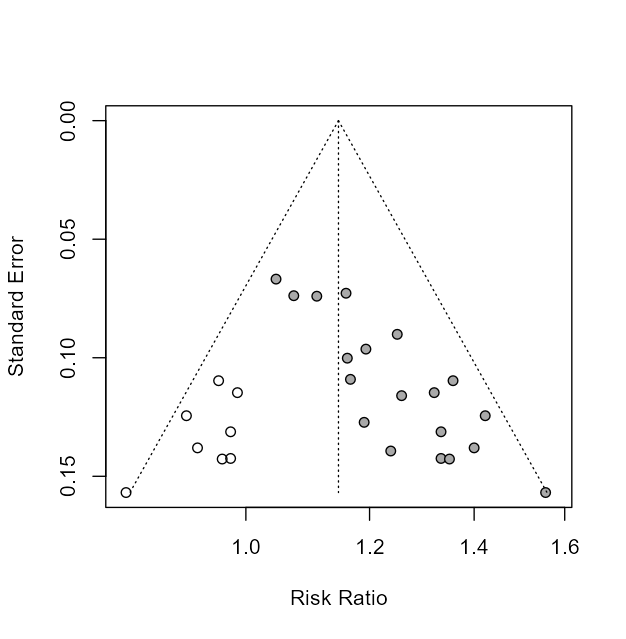


funnel plot of the clinical efficacy rate (after using trim-and-fill method)


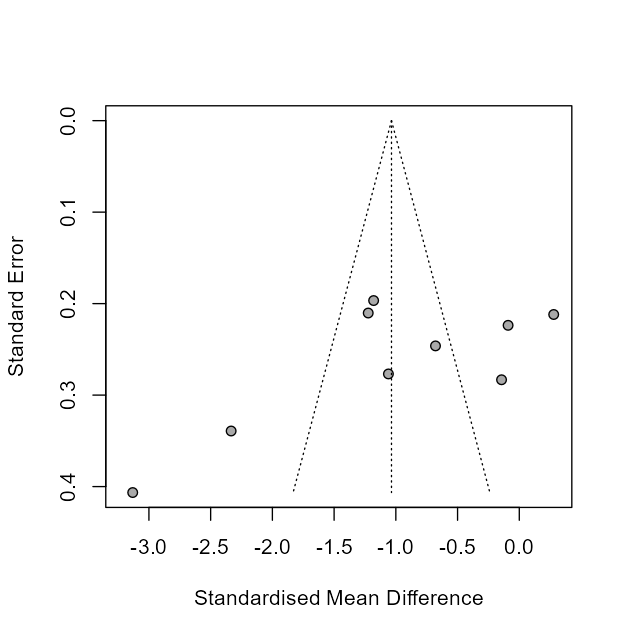


funnel plot of NT-proBNP


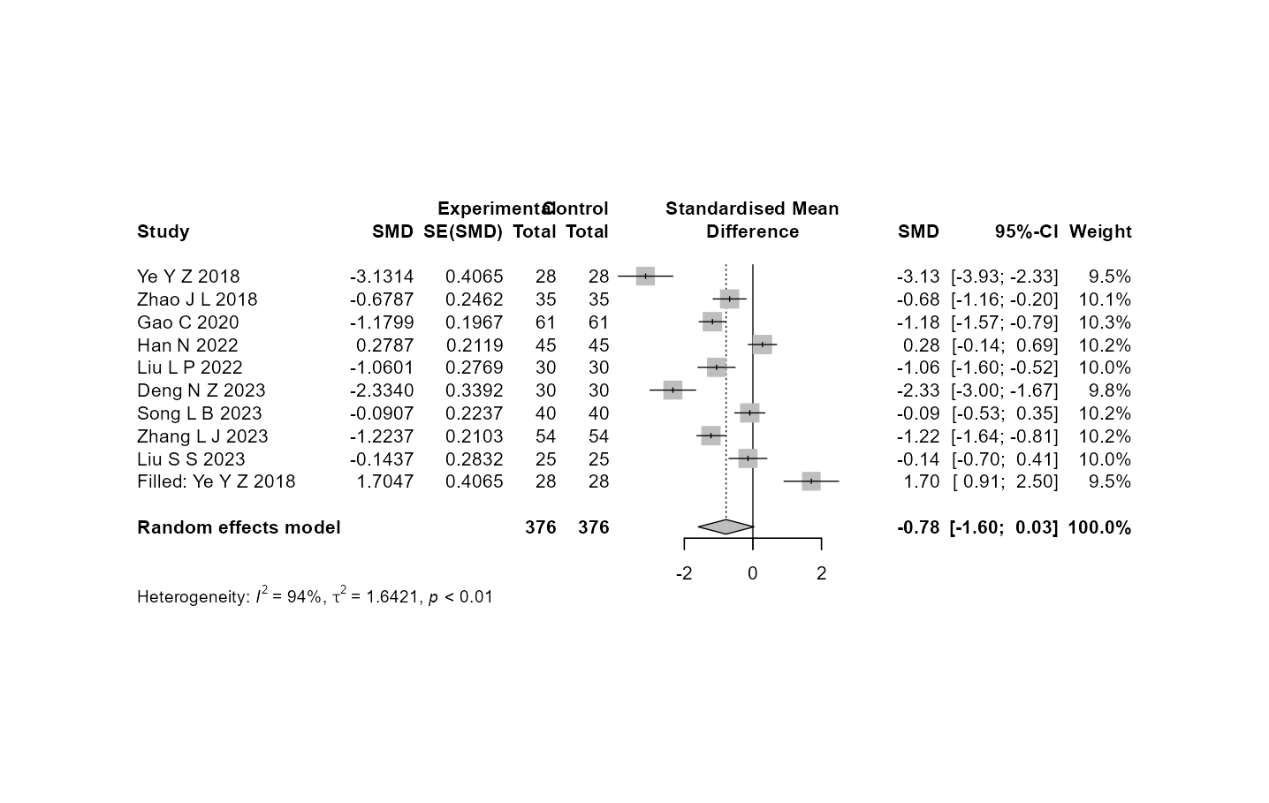


trim-and-fill method for NT-proBNP


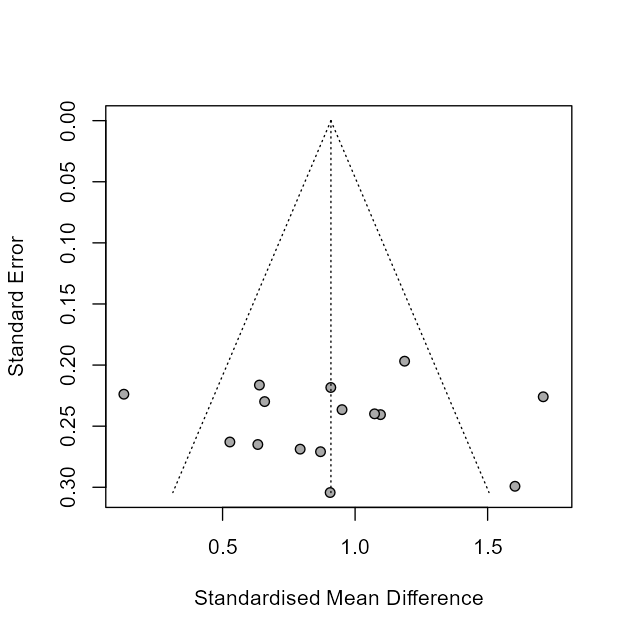


funnel plot of LVEF


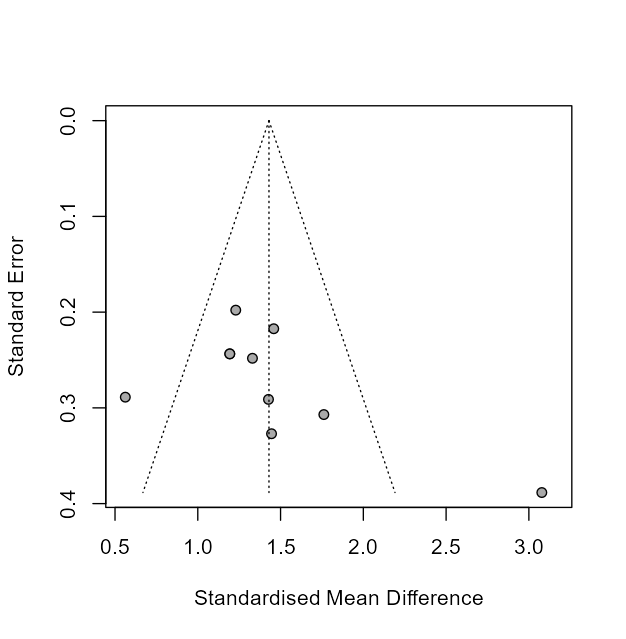


funnel plot of 6MWT
