# Supplementary material for: The effects of moxibustion in chronic heart failure patients: a systematic review and meta-analysis
Source: Front Cardiovasc Med. 2025 Jul 15;12:1552091. doi: 10.3389/fcvm.2025.1552091 (PMC12303964; doi:10.3389/fcvm.2025.1552091)
Supplement: Supplementary file 4 [file Table1.docx]

**Supplementary Table 1. Search strategy of PubMed**

| #1 heart failure [Mesh] |
| --- |
| #2 cardiac failure [Abstract/Title] OR myocardial failure [Abstract/Title] OR heart decompensation [Abstract/Title] OR HF [Abstract/Title] OR ventricular dysfunction [Abstract/Title] OR heart insufficiency [Abstract/Title] OR cardiac insufficiency [Abstract/Title] OR cardiomyopathies [Abstract/Title] |
| #3 1 OR 2 |
| #4 moxibustion [Mesh] |
| #5 moxabustion [Abstract/Title] |
| #6 #4 OR #5 |
| #7 #3 AND #6 |
